# Global Metabolomics of Fireflies (Coleoptera: Lampyridae) Explore Metabolic Adaptation to Fresh Water in Insects

**DOI:** 10.3390/insects13090823

**Published:** 2022-09-10

**Authors:** Linyu Yang, Zishun Zhao, Dan Luo, Mingzhong Liang, Qilin Zhang

**Affiliations:** 1 Faculty of Life Science and Technology, Kunming University of Science and Technology, Kunming 650500, China; 2 YEN, Chuxiong People’s Hospital, Chuxiong 675000, China; 3 Guangxi Key Laboratory of Marine Disaster in the Beibu Gulf, Ocean College, Beibu Gulf University, Qinzhou 535011, China

**Keywords:** aquatic firefly, metabolic profile, fresh water, metabolic adaptation

## Abstract

**Simple Summary:**

To date, little is known about the molecular mechanisms of aquatic adaptation in invertebrates, particularly in insects, which are the most abundant and diverse species. How aquatic insects adapt to freshwater environments remains largely unknown. Fireflies have terrestrial and aquatic lineages according to the habits of their larvae, and provides a good opportunity to explore aquatic adaptation of insects. We generated adult and larval metabolomes of two firefly species (aquatic *Aquatica leii* and terrestrial *Lychnuris praetexta*), and then a set of metabolites and their pathways involved in freshwater adaptation of aquatic firefly species were investigated by intraspecific and interspecific metabolomics comparisons, as well as by functional enrichment analysis. These molecules and pathways were primarily involved in oxidative stress/xenobiotics/immunity response, energy metabolism, sense function, and morphological attributes related to the freshwater lifestyle of aquatic *A. leii*. This study suggests that abundance-level changes in metabolites contributed to freshwater adaptation of fireflies, and provides insights into the metabolic mechanisms of aquatic adaptation in insects.

**Abstract:**

Aquatic insects are well-adapted to freshwater environments, but metabolic mechanisms of such adaptations, particularly to primary environmental factors (e.g., hypoxia, water pressure, dark light, and abundant microbes), are poorly known. Most firefly species (Coleoptera: Lampyridae) are terrestrial, but the larvae of a few species are aquatic. We generated 24 global metabolomic profiles of larvae and adults of *Aquatica leii* (freshwater) and *Lychnuris praetexta* (terrestrial) to identify freshwater adaptation-related metabolites (AARMs). We identified 110 differentially abundant metabolites (DAMs) in *A. leii* (adults vs. aquatic larvae) and 183 DAMs in *L. praetexta* (adults vs. terrestrial larvae). Furthermore, 100 DAMs specific to aquatic *A. leii* larvae were screened as AARMs via interspecific comparisons (*A. leii* vs. *L. praetexta*), which were primarily involved in antioxidant activity, immune response, energy production and metabolism, and chitin biosynthesis. They were assigned to six categories/superclasses (e.g., lipids and lipid-like molecules, organic acids and derivatives, and organoheterocyclic compound). Finally, ten metabolic pathways shared between KEGG terms specific to aquatic fireflies and enriched by AARMs were screened as aquatic adaptation-related pathways (AARPs). These AARPs were primarily involved in energy metabolism, xenobiotic biodegradation, protection of oxidative/immune damage, oxidative stress response, and sense function (e.g., glycine, serine and threonine metabolism, drug metabolism-cytochrome P450, and taste transduction), and certain aspects of morphology (e.g., steroid hormone biosynthesis). These results provide evidence suggesting that abundance changes in metabolomes contribute to freshwater adaptation of fireflies. The metabolites identified here may be vital targets for future work to determine the mechanism of freshwater adaptation in insects.

## 1. Introduction

Compared with terrestrial habitats, fresh water possesses a relatively lower content of oxygen (hereafter referred to as hypoxia) and light, water pressure, as well as more abundant pathogenic bacteria communities [1,2]. These factors have greatly affected the evolution of aquatic animals and their ecological strategies. In long-term adaptation, aquatic animals have undergone significant changes in morphology, behavior, and physiology, especially regarding respiratory patterns and swimming style [3,4]. So far, the existing studies on aquatic adaptation of animals have focused on vertebrates, particularly cetaceans (whales, dolphins, and porpoises) [5]; others include loons [6], soft shell turtle (Trionychidae) [7], fish [8,9], and marine invertebrates such as corals and green crabs [10,11]. Overall, these investigations focused on aquatic adaptations of animals regarding behavior, morphology, physiology, and genetic/molecular basis (DNA and RNA); however, the exploration of the metabolic mechanisms behind aquatic adaptation is lacking.

Despite the accumulation of metabolite information and the wide use of mass-spectrometric techniques in metabolomics, only a few studies have explored the metabolic mechanisms underlying aquatic adaptations of animals. For example, body color is an important ecological trait in aquatic animals, influencing their survival [11,12]. Metabolomics analysis of body color formation in the leopard coral grouper (*Plectropomus leopardus*) showed greater melanin synthesis activity in black compared to red-colored groups [11]. When tambaqui juveniles (*Colossoma macropomum*) faced hypoxia, anaerobic metabolism rapidly adjusted glucose and lactate metabolism, providing adequate energy [13]. Overall, little is known about the metabolic mechanisms of aquatic adaptation in insects as the most abundant and diverse animal.

Aquatic insects (defined by having at least one aquatic life stage) make up 80% of total aquatic animal diversity [1,14]. Most aquatic insects inhabit freshwater environments and suffer from more extreme physiological challenges (e.g., hypoxia, water pressure, and diminished light) than terrestrial insects [1]. Compared to their terrestrial counterparts, aquatic insects present adaptive morphological characteristics, such as a streamlined dorsum, a flattened and smooth body, and a closed tracheal system with air bubbles and gills for oxygen intake [14]. Moreover, the developmental transition from aquatic larvae to terrestrial adults involves several physiological performance-related changes, such as increased flight muscle performance in odonates [15] and sodium chloride (NaCl) transport across the cuticle in *Aedes* mosquitoes [16]. This implies that metabolic changes are key to freshwater adaptation of aquatic insects, and indicates different metabolic mechanisms between aquatic and terrestrial groups.

The Lampyridae family (Insecta: Coleoptera: Cantharoidae), commonly known as fireflies, includes at least 2000 recorded species belonging to 100 genera, widely distributed and found in tropical, subtropical, and temperate zones [17,18]. They undergo complete metamorphosis. Their developmental stages include eggs, larvae, pupae, and adults. According to larval habitat preferences, firefly species can be divided into terrestrial (e.g., genera *Asymmetricata*, *Lychnuris* and *Pteroptyx*), aquatic (*Luciola* and *Aquatica*), and semi-aquatic lineages (*Pygoluciola*) [19]. Among them, terrestrial groups contain the vast majority of species (more than 98%). Moreover, compared to terrestrial and semi-aquatic firefly larvae, aquatic species exhibit adaptations to freshwater environments in larval morphology (e.g., branched tracheal gills and both smooth and soft bodies) and behaviour (e.g., swimming) [19,20]. In recent years, comparative transcriptomic analysis of fireflies has been conducted to explore their molecular adaptation to freshwater environments [14]. Genome-scale evolutionary constraints, adaptive signals to the freshwater environment in the sequence and expression levels of candidate genes related to ATP metabolism, immune and hypoxia responses, and insect-specific morphology have all been uncovered in aquatic fireflies. The above uncovered morphological, behavioural, genetic characteristics indicate that aquatic firefly species (Lampyridae) are a suitable model for investigating freshwater adaptation of insects. Currently, despite investigations on adaptive behavior, molecular basis, and morphology of the aquatic firefly have been conducted, metabolic mechanisms remain largely unknown. Even when extended to other insect groups, few studies have explored insects’ adaptation to water environment at a metabolomics level. Fireflies provide an opportunity to explore the metabolic adaptations of insects to freshwater environments.

Here, we sequenced and intraspecifially and interspecifically compared the adult and larval metabolic profiles of two firefly species (aquatic *Aquatica leii* and terrestrial *Lychnuris praetexta*). Then, metabolites with significant changes in *A. leii* were identified by comparing differentially abundant metabolites (DAMs) between both species. Finally, functional analyses (e.g., category division and metabolic pathway enrichment) were performed on candidate metabolites involved in freshwater adaptations.

## 2. Materials and Methods

### 2.1. Insect Materials

**Figure 1:**
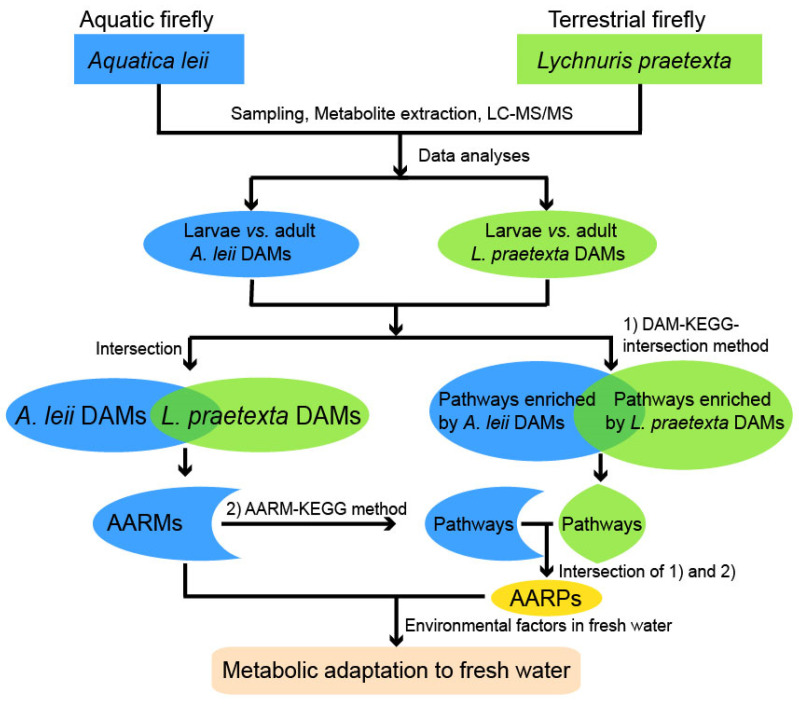
Flow chart of experiments and data processes. Aquatic fireflies have an aquatic larval stage and a terrestrial adult stage; both larvae and adults of terrestrial fireflies are terrestrial. DAMs: differentially abundant metabolites. KEGG: Kyoto Encyclopedia of Genes and Genomes. AARMs: aquatic adaptation-related metabolites. AARPs: aquatic adaptation-related pathways. DAM-KEGG-intersection and AARM-KEGG methods described in detail in Section 2.6.

Larvae (4th–6th instars) and adults of *A. leii* and *L. praetexta* were raised as previously described (Supplementary information in Zhang et al., 2020). *A. leii* freshwater larvae (ALL) and terrestrial adults (ALA) were randomly collected, to a total of 50 4th instar, 50 5th instar, and 30 6th instar larvae, and 20 female and 20 male adults. We additionally randomly collected larvae (LPL) and adults (LPA) of *L. praetexta* inhabiting terrestrial environments across the entire life cycle, to a total of 10 4th instar, 6 5th instar, and 5 6th instar larvae, and 20 female and 20 male adults. Each collected sample per developmental stage was put in a 10 mL plastic centrifuge tube and then stored at −80 °C. The sampling was independently repeated six times as six biological replicates. The intestines were removed from all individuals to avoid disturbance of residual foods and gut microbes. A flow diagram of the experiments and data analyses is shown in Figure 1.

### 2.2. Metabolite Extraction

For metabolite extraction of each replicate, 50 mg of firefly tissue at each developmental stage was mixed with 400 μL of pre-cooled extracting solution (methanol:water = 3:1, V/V) in a 2 mL Eppendorf tube, and then a 6 mm diameter grinding bead was added. After grinding for 6 min at 50 Hz at 10 °C in a Wonbio-96c high-throughput tissue crusher (Wanbo Biotechnology, Shanghai, China), the samples were extracted at 4 °C at 40 kHz for 30 min using a controlled-temperature ultrasonic cleaning machine (SBL-10TD, Scientz Biotechnology, Ningbo, China). The samples were placed at −20 °C for 10 min for proteins to precipitate. After centrifugation at 30,000× *g* at 4 °C for 15 min (refrigerated centrifuge 5430R; Eppendorf, Hamburg, Germany), the supernatant of each developmental stage of 4th–6th instars of *A. leii* samples was equally mixed as ALL samples and used for further global metabolomics analysis. Adult *A. leii*, 4th–6th instars of *L. praetexta*, and adult *L. praetexta* samples were also similarly processed as ALA, LPL, and LPA samples, respectively. In total, the samples were used to generate 24 metabolic profiles (i.e., ALL_1–6, ALA_1–6, LPL_1–6, and LPA_1–6).

In addition, to avoid systematical and technical errors caused by metabolome sequencing, 5 µL from each collected supernatant sample was mixed into a 2 mL Eppendorf tube as a quality control sample (QC; *n* = 3). The QC samples were sequenced parallel to the experimental samples, which represented the whole sample set, and were injected at regular intervals to monitor stability in the sequencing process.

### 2.3. Global Metabolomics Analysis

Global metabolomics analysis was implemented using liquid chromatography with tandem mass spectrometry (LS-MS/MS) as previously described [21], using contained an ultrahigh performance liquid chromatography (UHPLC) system (Vanquish, Thermo Fisher Scientific, Waltham, MA, USA) with a UPLC HSS T3 column (100 mm × 2.1 mm, 1.8 µm) coupled to a Q Exactive HF-X mass spectrometer (Orbitrap MS, Thermo Fisher Scientific). The gradient elution parameters were set as follows: 0–3.5 min: 100% phase A (95% water/5% acetonitrile + 0.1% formic acid), 0.4 mL/min (mobile phase velocity); 3.5–5 min: 75.5% A–24.5% B (5% water/47.5% acetonitrile/47.5% isopropanol + 0.1% formic acid), 0.4 mL/min; 5–5.5 min: 35% A–65% B, 0.4 mL/min; 5.5–7.4 min: 100% B, 0.4 mL/min; 7.4–7.6 min: 100% B, 0.6 mL/min; 7.6–7.8 min: 48.5% A–51.5% B, 0.6 mL/min; 7.8–9 min: 100% A, 0.5 mL/min; 9–10 min: 100% A, 0.4 mL/min. The auto-sampler temperature was set to 4 °C, and the injection volume was 2 μL. The range for *m/z* detection was 70∼1050. Q Exactive HF-X mass spectrometer was used for its ability to acquire MS/MS spectra in information-dependent acquisition (IDA) mode, controlled by the acquisition software (Xcalibur, Thermo Fisher Scientific). In this mode, the acquisition software continuously evaluates the full scan MS spectrum. The electrospray ionization (ESI) source conditions were set as follows: sheath gas flow rate of 50 arb, aux gas flow rate of 13 arb, capillary temperature 325 °C, normalized collision energy 204,060 eV, full MS resolution at 60,000, resolution (MS/MS) at 7500, collision energy at 10/30/60 in normalized collisional energy (NCE) mode, and spray voltage at (+) 3.5 kV or (–) 3.5 kV. Raw data of the 24 metabolic profiles of ALL, ALA, LPL, and LPA (*n* = 6) were obtained by collecting data in positive and negative ion modes.

### 2.4. Data Preprocessing

The raw data were converted into the mzXML format by using ProteoWizard, and processed using R package XCMS (version 3.2) for peak detection, extraction, alignment, and integration, as previously described [21,22]. The software generated a data matrix for the raw data, including sample information, retention time (RT), mass-to-charge ratio (*m/z*) values, and peak intensity. Metabolic features that were detected at least in 70% of samples were retained. After filtering, minimum metabolite values were assessed for samples in which the metabolite levels fell below the lower limit of quantitation. Peak annotation was carried out using Compound Discover v3.0 (Thermo Fisher Scientific) and OSI-SMMS v1.0 (software system for rapid identification and analysis of small molecular compounds in metabolomics; Dalian Chem Data Solution Information Technology, Dalian, China), integrated with the mzcloud database (HighChem LLC, Bratislava, Slovakia). An internal standard was used for data QC (reproducibility). Metabolic features with relative standard deviation (RSD) of QC > 30% were discarded. Following normalization procedures and imputation, statistical analysis was performed on *log*-transformed data to identify significant differences in metabolite levels between comparable groups. Subsequently, the acquired MS/MS spectra were matched against metabolome databases including HMDB (Hydrogen Mitigation Design Basis, http://www.hmdb.ca/, accessed on 28 April 2022), KEGG (Kyoto Encyclopedia of Genes and Genomes, http://www.genome.jp/kegg/, accessed on 28 April 2022), and an in-house MS/MS database (Biotree Biotech Co., Ltd. Shanghai, China). The MS/MS spectra match score was calculated using a dot-product algorithm and ranged from 0 to 1. The cutoff for match score was set as 0.4. The data were normalized by the internal standards before statistical analysis. The Pearson correlation coefficient was calculated between each sample pair to evaluate the repeatability of the biological replicates. Principal Component Analysis (PCA) was performed to separate and classify the sample groups based on metabolic profiles using SIMCA-*P*+ software v16 (Sartorius Stedim Data Analytics AB, Umea, Sweden).

### 2.5. Identification of Differential Abundant Metabolites (DAMs)

The significance of each DAM between larvae and adults of each firefly species was evaluated using unpaired Wilcoxon tests. The *p*-values were further corrected by the Benjamini-Hochberg adjustment method (false discovery rate, *FDR* < 0.05), |log2FC(fold change)| > 1, and VIP (the variable importance of projection) > 2. The metabolites involved in freshwater adaptation of larval *A. leii* were detected as previously described (Zhang et al., 2020). Briefly, the DAMs of *L. praetexta* were intersected with that of *A. leii*, thus excluding non-responding metabolites to fresh water in larval *A. leii*. Subsequently, DAMs that were only detected in *A. leii* (*A. leii*-specific DAMs) were retained as metabolites that presented abundance changes to freshwater environments; these metabolites were identified as aquatic adaptation-related metabolites (AARMs) in *A. leii* larvae.

### 2.6. HMDB Classification and KEGG Functional Enrichments

According to the HMDB database annotation information, AARMs were divided and assigned to three levels: superclass, class, and subclass. In addition, the identified differential metabolites were further analyzed to determine the relevant biological pathways using MetaboAnalyst 5.0 (http://www.metaboanalyst.ca/, accessed on 3 May 2022). Benjamini-Hochberg adjustment was employed as the FDR correction method. To detect reliable KEGG pathways specifically involved in freshwater adaptation of larval *A. leii*, two methods (i.e., DAM-KEGG-intersection and AARM-KEGG) were used, as previously described (Zhang et al., 2020). In the DAM-KEGG-intersection method DAMs of *L. praetexta* and *A. leii* were subjected to KEGG enrichment analysis, their results were intersected, and the pathways specific to *A. leii* were obtained. In the AARM-KEGG method, the AARMs above obtained were directly used for KEGG enrichment. The results shared by both methods were considered as the final KEGG metabolic pathways associated with freshwater adaptation of *A. leii* (namely, aquatic adaptation-related pathways, AARPs).

## 3. Results

### 3.1. Overview of Sequencing Data

**Figure 2:**
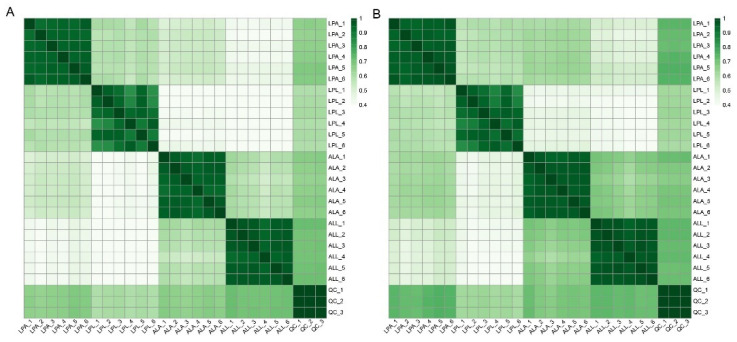
(**A**,**B**) correlation heat map among samples of *A. leii* larvae (ALL), *A. leii* adults (ALA), *L. praetexta* larvae (LPL), *L. praetexta* adults (LPA) and quality control (QC) in ESI+ and ESI−.

**Figure 3:**
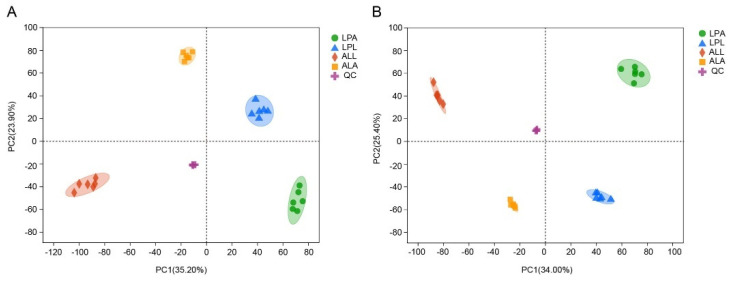
PCA scatter plot scores in ESI+ (**A**) and in ESI− (**B**) scan modes for the first two components of five groups (ALL, ALA, LPL, LPA, and QC).

A total of 26,140 peaks (13,643 and 12,497 peaks in positive (ESI+) and negative (ESI−) ion mode, respectively) were detected by the mass spectrometer. Through further screening, 20,276 peaks (10,433 peaks in ESI+ and 9843 peaks in ESI−) were obtained. By further screening peaks, a total of 2928 metabolites (2030 metabolites in ESI+ and 938 metabolites in ESI−) were identified. The correlation value was close to 1 in the ESI+ (Figure 2A) and ESI− (Figure 2B), indicating robust sampling and metabolome sequencing. The PCA results showed cluster samples in each group, and a clear separation among five groups (including four experimental and one QC groups), both in the ESI+ (Figure 3A) and ESI− (Figure 3B), suggesting significant differences of metabolite profiles among the five groups.

### 3.2. Analysis of Aquatic Adaptation-Related Metabolites (AARM) in A. leii Larvae

**Figure 4:**
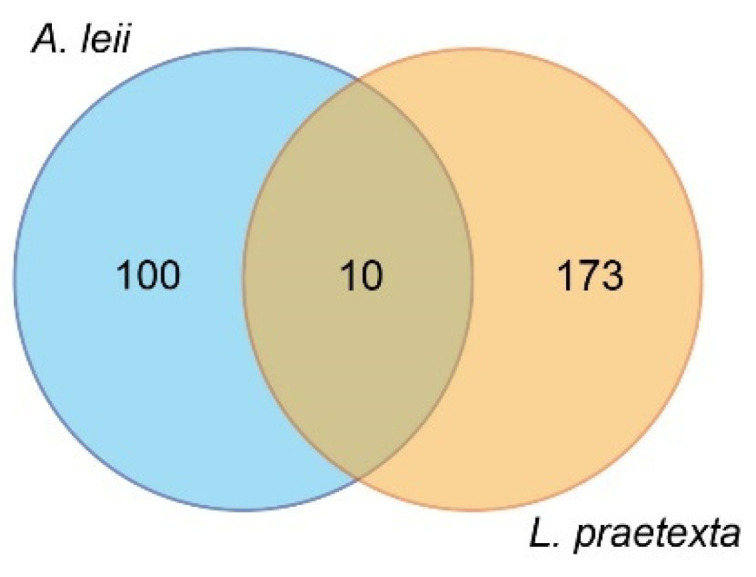
Venn diagram of the number of DAMs shared by *A. leii* and *L. praetexta*.

In total, 100 AARMs were identified (Figure 4). According to their annotation information and references (Table 1), 17 AARMs, including 15 up- and 2 down-regulated metabolites, were classified into functional categories involving their antioxidant activity. A total of 22 AARMs (17 up- and 5 down-regulated) were identified to be associated with immunity; in particular (some of them cover multiple immune functions), 10 metabolites were involved in anti-inflammatory response (e.g., 12a-hydroxy-3-oxocholadienic acid, 21-deoxycortisol, and 3-formyl-6-hydroxyindole), 10 metabolites involved in anticancer response (e.g., capsianoside H, 5′-deoxy-5-fluorocytidine, and glutamylproline), four metabolites involved in antibacterial response (e.g., agavoside G, annoglabasin F, and 2,3-Dihydroabscisic alcohol), two metabolites involved in antiviral response (1-(3-Furanyl)-6,7-dihydroxy-4,8-dimethyl-1-nonanone and 23-trans-p-Coumaroyloxytormentic acid), two metabolites involved in immune responses (2-hydroxyestrone sulfate and 19-oxoandrost-4-ene-3,17-dione), and two metabolites involved in immunomodulatory effects (Canarigenin 3-[glucosyl-(1->4)-6-deoxy-alloside] and Dynorphin A (6–8)). In addition, 18 AARMs were related to energy metabolism, 15 of which showed up-regulation, while three were down-regulated. Two AARMs were typically related to chitin biosynthesis in response to freshwater environments, including (2E,8Z)-Decadiene-4,6-diyn-1-yl-3-methylbutanoate and 25-Hydroxytachysterol3. The exact biological function of the 41 remaining AARMs identified in this study was not reported in previous publications.

### 3.3. HMDB Class Analysis of Aquatic Adaptation-Related Metabolites (AARM) in A. leii Larvae

**Figure 5:**
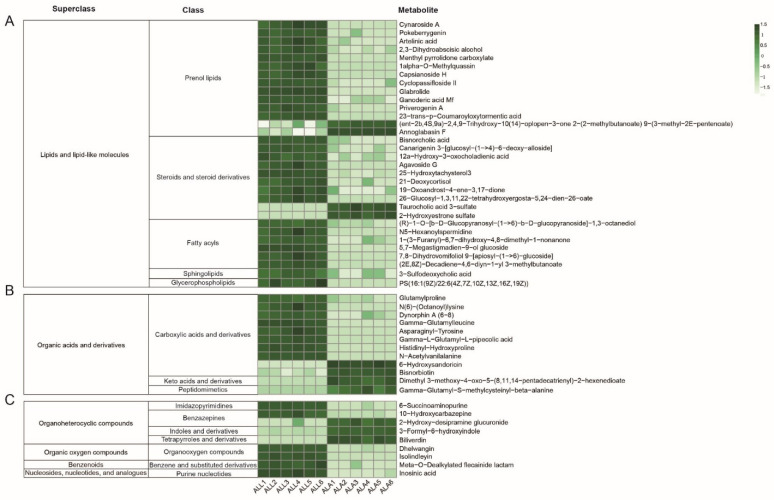
Heat maps of differentially abundant metabolites between *A. leii* larvae and adults. (**A**) Metabolic levels of AARM contained in lipids and lipid-like molecules; (**B**) Metabolic levels of AARM contained in organic acids and derivatives; (**C**) Metabolic levels of AARM contained in organoheterocyclic compounds, organic oxygen compounds, benzenoids, and nucleosides, nucleotides, and analogues. Each line represents a differentially abundant metabolite, and each row represents a sample. Different colors represent different abundances, with darker colors indicating higher abundance.

In total, 53 AARMs were annotated in the HMDB database (Table S4), and were divided into six superclasses (Figure 5), including lipids and lipid-like molecules, organic acids and derivatives, organoheterocyclic compounds, organic oxygen compounds, benzenoids, and nucleosides, nucleotides, and analogues. At the class level, the lipids and lipid-like molecules included prenol lipids, steroids and steroid derivatives, fatty acyls, sphingolipids, and glycerophospholipids (Figure 5A). A total of 32 AARMs belonged to the lipid and lipid-like molecules superclass; most of them belonged to the prenol lipids class, with 12 up-regulated and 2 down-regulated metabolites. For steroids and steroid derivatives, 10 AARMs (i.e., eight up- and two down-regulated metabolites) were included. All the six AARMs belonging to the fatty acyl class showed up-regulation. The sphingolipids and glycosphingolipids classes identified only one member each, presenting up-regulation abundance in response to fresh water in aquatic *A. leii* larvae. Within the six superclasses, organic acids and derivatives contained the second largest number (12) of AARMs. This superclass included carboxylic acids and derivatives (10 ARMS, from which eight were up- and two were down-regulated), keto acids and derivatives, and peptidomimetics (Figure 5B). The last two categories included only one member each, presenting down-regulation abundance. Regarding the other four superclasses (Figure 5C), organoheterocyclic compounds included imidazopyrimidines (1 up-regulated AARM), benzazepines (1 up- and 1 down-regulated AARM), indoles and deriva (1 down-regulated AARM), and tetrapyrroles and derivatives (1 down-regulated AARM). In organic oxygen compounds superclass, one class—organooxygen compounds—included two up-regulated AARMs. For the benzenoids and the nucleosides, nucleotides, and analogues superclasses, each covered only one class: benzene and substituted derivatives, and purine nucleotides, respectively. All the AARMs included in these two superclasses were up-regulated in aquatic *A. leii* larvae.

### 3.4. Pathway Analysis of Aquatic Adaptation-Related Metabolites (AARM) in A. leii Larvae

**Figure 6:**
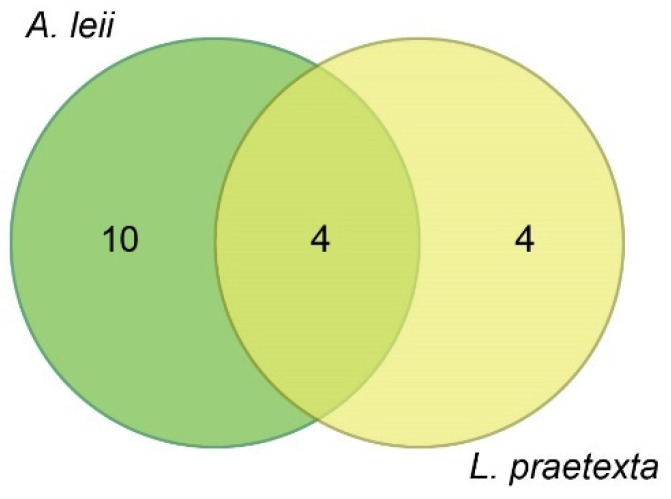
Venn diagram of KEGG terms enriched by DAMs between larvae and adults from *A. leii* and *L. praetexta*.

Through the DAM-KEGG-intersection method described to detect reliable KEGG pathways specifically involved in freshwater adaptation of larval *A. leii*, we observed that DAMs (aquatic larvae vs. adults) were significantly enriched to 14 KEGG pathways (Table S5), such as antifolate resistance (map01523), riboflavin metabolism (map00740), and taste transduction (map04742). Meanwhile, DAMs in *L. praetexta* (terrestrial larvae vs. terrestrial adults) were significantly enriched to eight KEGG pathways (Table S6), such as insect hormone biosynthesis (map00981), folate biosynthesis (map00790), and histidine metabolism (map00340). Furthermore, 10 KEGG pathways specific to *A. leii* were identified (Figure 6), such as antifolate resistance (map01523), riboflavin metabolism (map00740), and taste transduction (map04742) (Table 2). In addition, via the AARM-KEGG method we applied, all the above obtained AARMs were enriched to 13 metabolic pathways, such as antifolate resistance (map01523), arachidonic acid metabolism (map00590), and biosynthesis of cofactors (map01240) (Table S7). Finally, the results of intersection analysis showed that all 10 metabolic pathways obtained by the DAM-KEGG-intersection method were included in the results obtained by the AARM-KEGG method (Figure S1). Both methods obtained 10 consistently enriched pathways, which were identified as target pathways (AARPs) involved in freshwater adaptation of *A. leii* (Table 2).

## 4. Discussion

### 4.1. Metabolite Function Linked to Freshwater Adaptation

Antioxidants protect animal cells against DNA and cell damage, cytotoxicity, and apoptosis under hypoxia [58] by reversing the increase in reactive oxygen species (ROS) that can occur in circumstances such as water hypoxia that trigger more ROS and toxicity than in terrestrial environments [59]. Overall, in the present study, most antioxidants were found to be up-regulated in aquatic *A. leii* larva compared to adults, likely a mechanism for ROS elimination resulting from hypoxia. In particular, several oligopeptides (2~10 amino acids) detected in this study possess antioxidant activity [23], such as those generated by hydrolysis of porcine collagen [60] and isolated from skipjack tuna (*Katsuwonus pelamis*) dark muscle [61]. In addition, pokeberrygenin and priverogenin A as triterpenoids were ROS modulators of cells [26]. Cynaroside A is a flavonoid with proven antioxidant activity, contributing to the adaptation of the Tibetan sheep to high-altitude cold and hypoxia [25]. Treatments with both low and high amounts of inosinic acid eliminated mouse lumenal oxidative stress [27]. The increasing abundance of these four metabolites likely contributed to the elimination of ROS generated by hypoxia under fresh water. Energy metabolism and antioxidation jointly regulated the response of organisms to environmental stress, as previously reported in *Strigomonas culicis* [62] and in large yellow croaker (*Larimichthys crocea*) [63]. This indicates that abundance changes of antioxidation-related metabolites accompanied by energy metabolism contributed to scavenge ROS. This is useful for the maintenance of a normal ROS level, assisting in the adaptation of *A. leii* larvae to hypoxia in fresh water. 

In addition, the animal immune response is highly sensitive to abiotic and biotic stresses such as hypoxia, water pressure, and water pollutants/microbes. For example, tissue oxygen deficit caused by the exogenous pollutant fipronil, an important commonly used insecticide, could induce nonspecific immunity in common carp (*Cyprinus carpio*) [64], and the immune efficacy of *Eogammarus posjeticus* significantly changed under various water pressures [65]. When fishes (e.g., *Odontesthes bonariensis*, *Oncorhynchus mykiss*, *Oryzias melastigma*) were exposed to organic pollutants, the metabolism of 6-succinoaminopurine significantly changed to enhance immune defense [39]. Canarigenin 3-[glucosyl-(1->4)-6-deoxy-alloside] belonging to saponin has been found to have anti-inflammatory and antioxidant functions, and to affect cell membrane permeability [42]. In the present study, many AARMs were found to be involved in immune functions, most of which showed up-regulation. Therefore, these immune-related AARMs probably contributed to the adaptation of aquatic firefly to hypoxia, water pressure, and pollutant exposure/microbial invasion in fresh water. Meanwhile, energy metabolism was essential for the antimicrobial response, elimination of inflammation and injured cells, and other biological functions [66]. Previous studies found a balance between energy production and metabolism to be involved in the response of animals to hypoxia and pressure in water [67,68]. In particular, biliverdin is an important metabolite that participates in mitochondrial energy consumption [53], while the oligopeptides Thr-Val-Val and Val-Leu-Ser, and gamma-L-glutamyl-L-pipecolic acid are involved in ATP production [51]. Bisnorcholic acid participates in energy metabolism and antibacterial and immune responses [32]. In the current study, several AARMs were found to be related to energy production and consumption, which might be key for maintaining the dynamic balance of energy in freshwater adaptation of aquatic firefly, as similarly reported from transcriptomics perspectives [14].

Cuticle chitin biosynthesis (including the chitin-containing exoskeleton) contributes to an adaptation to the increasing water pressure and both the size and morphology of the open tracheal system (the chitin-containing lining of the tracheal tube) in insects [14]. In this study, two detected AARMs were related to insect-specific freshwater adaptation morphology. Namely, (2E,8Z)-Decadiene-4,6-diyn-1-yl-3-methylbutanoate and 25-Hydroxytachysterol3 participate in cuticle formation [56,57]. This result suggests that these two AARMs may have contributed to the required adaptation in respiration, movement, and resistance to water pressure of aquatic firefly. In addition, tyrosinase inhibitor (isolindleyin) and tyrosine (6-hydroxysandoricin) impact melanin synthesis [45]. In this study, the increased isolindleyin and decreased 6-hydroxysandoricin impacted melanin synthesis involved in light response, which may be an adaptive characteristic of aquatic firefly to low light under water.

### 4.2. HMDB Categories Linked to Freshwater Adaptation

More than half of AARMs were assigned to various categories at different levels, according to the HMDB database. Lipids and lipid-like molecules included the largest number of AARMs, and most of them were up-regulated in aquatic *A. leii* larvae compared with adults. Previous studies reported a greater amount of lipids and lipid-like molecules in aquatic than in terrestrial insects [69]. In general, lipids and lipid-like molecules are structural components of the membrane [70], and ca affect the morphological structure and composition of cell membranes involved in tolerance against water pressure [71]. Abundance change of lipids and lipid-like metabolites may be related to the adaptation of *A. leii* larvae to water pressure.

Interestingly, steroids and steroid derivatives belonging to lipids and lipid-like molecules have been widely found in aquatic crustaceans, some of which act as hormones used to regulate the molting of arthropods [72]. AARMs involved in steroids and steroid derivatives may have facilitated the formation of a suitable chitin-containing exoskeleton, contributing to an adaptation to water pressure in *A. leii*. Organic acids are intermediate products of metabolism, and play important roles in various cellular biochemical pathways, such as energy metabolism and detoxification [73]. A study reported that the metabolism of organic acids in *Callosobruchus chinensis* larvae increased when exposed to hypoxia [74]. In addition, several studies proved that carboxylic acids and derivatives, keto acids and derivatives, and peptidomimetics present antibacterial and anti-inflammatory activity [75,76,77]. Therefore, an altered metabolism of organic acids and derivatives contribute to hypoxia adaptation and immune responses of *A. leii* in fresh water. In addition, nucleosides, nucleotides, and analogues are a diverse group of endogenous metabolites widely present in organisms [78]. DNA can be more easily damaged under water than terrestrial environmental stress (e.g., water pressure and hypoxia) [79]. In the present study, the increasing abundance of AARMs assigned to nucleosides, nucleotides, and analogues likely improved the ability of aquatic firefly to efficiently repair DNA. In addition, a previous study reported an increased abundance of organic oxygen compounds in *Anopheles sinensis* larvae under deltamethrin exposure [80], and organoheterocyclic compounds were described as immunomodulatory metabolites in yogurt [81]. AARMs categorized as organic oxygen and organoheterocyclic compounds in the current study likely contributed to freshwater adaptation of aquatic firefly by adjusting immune-related and toxic-related metabolism.

### 4.3. Metabolic Pathways Linked to Freshwater Adaptation

Metabolites belonging to the glycine, serine, and threonine metabolism pathway significantly changed in *Callosobruchus chinensis* larvae when exposed to hypoxia [74]. Together with the antioxidant function of this pathway [82], the glycine, serine, and threonine metabolic pathway may have enhanced the antioxidant capacity of aquatic firefly tissues under freshwater hypoxia. The antifolate resistance pathway, one AARP identified in this study, was reported to be significantly changed in high-altitude adaptation of Tibetans, adjusting folate metabolism [83], also contributing to differences in the metabolic rate of folic acid at different oxygen concentrations [84]. Moreover, riboflavin supplementation improved energy metabolism efficiency in mice exposed to acute hypoxia [85]. The antifolate resistance pathway thus endorsed hypoxia adaptation of *A. leii* larvae in freshwater environments by promoting riboflavin-mediated energy metabolism. In addition, purine metabolism is an important pathway in mammalian oxidative stress adaptation [86]. For example, an increased capacity for purine recycling and ATP synthesis from inosine monophosphate was detected in ischemic tissues of bottlenose dolphins (*Tursiops truncatus*) during diving [87]. In the present study, purine metabolism was identified as an AARP, indicating its key role in oxidative stress adaptation of not only mammals but insects, via increased purine utilization and ATP production. Hypoxia induced ROS in male rats with an immune response, while antioxidant biotin (e.g., vitamin E) could block this increase and avoid immune injury [88]. Biotin metabolism, here identified as an AARP, expanded a key function of biotin as protectant of oxidative/immune damage in freshwater adaptation of animals. In addition, drug metabolism-cytochrome P450, identified as an AARP, and a well-documented KEGG term involving xenobiotic biodegradation and metabolism, responded to the benzo(a)pyrene stress in *A. leii* larvae [89], suggesting that detoxification-related pathways probably regulate hypoxia adaptations of aquatic fireflies by balancing oxygen use. 

Several studies have shown differences in olfaction triggered by chemosensory-related genes (e.g., ionotropic receptors involved in smell and taste) between terrestrial Lepidoptera and aquatic Trichoptera insects [90]. Indeed, the taste transduction pathway was identified as an AARP in *A. leii* in the present study. This evidence shows the importance of metabolic pathways involved in the olfaction and taste functions for freshwater adaptation of insects. In addition, as a key pathway that impacted the timing of molting and metamorphosis of insects [91], the steroid hormone biosynthesis pathway was found to be involved in freshwater adaptation in *A. leii*. This reflects how the aquatic lifestyle of insects may benefit from adaptive changes in timing of molting and metamorphosis.

## 5. Conclusions

This study explored the metabolic mechanisms regarding adaptation of insects to freshwater environments using global metabolomic profiling of the Lampyridae family. We documented candidate adaptive changes in the metabolites involved in antioxidant activity, immunity, energy metabolism, and morphological attributes (e.g., exoskeleton and the tracheal system) related to the freshwater lifestyle. These metabolites were assigned to categories linked to responses to freshwater environmental stressors (e.g., hypoxia, water pressure, limited light, and the abundant microbes). Furthermore, we also identified metabolic pathways that likely contribute to firefly freshwater adaptation. The single species used for freshwater and terrestrial fireflies in this study likely limited the applicability of information. Thus, to expand the base of evidence supporting the metabolite candidates obtained here, it is important to sequence metabolic profiling of more firefly species in the future.

## Figures and Tables

**Table 1:** Aquatic adaptation-related metabolites (AARMs) with function information. #: According to references.

Function	Specific Functions #	Id	Metabolites	Log2FC(ALL/ALA)	FDR	References
Antioxidants	Anti-Oxidant	metab_23671	Asparaginyl-Tyrosine	2.64	6.82 × 10^−3^	[23]
	Anti-Oxidant	metab_9504	Cinchonidine	1.41	7.84 × 10^−3^	[24]
	Anti-Oxidant	metab_17388	Cynaroside A	3.66	6.82 × 10^−3^	[25]
	Anti-Oxidant	metab_18889	Leu-Asp-Glu-Lys	1.78	6.82 × 10^−3^	[23]
	Anti-Oxidant	metab_22119	Lys-Gln-Asp-Lys	8.56	6.82 × 10^−3^	[23]
	Anti-Oxidant	metab_2651	Lys-Glu-Ser-Leu-Ser	1.37	7.84 × 10^−3^	[23]
	Active oxygen regulator	metab_6098	Pokeberrygenin	1.85	7.84 × 10^−3^	[26]
	Active oxygen regulator	metab_8499	Priverogenin A	1.69	7.84 × 10^−3^	[26]
	Anti-Oxidant	metab_10795	Tyr-Glu-Asp	−1.60	7.84 × 10^−3^	[23]
	Anti-Oxidant	metab_9513	Tyr-Phe-Glu	1.90	7.84 × 10^−3^	[23]
	Anti-Oxidant	metab_2367	Tyr-Pro-Trp	1.88	7.84 × 10^−3^	[23]
	Anti-Oxidant	metab_11607	Val-His-Tyr-Tyr	3.07	7.84 × 10^−3^	[23]
	Anti-Oxidant	metab_25229	Inosinic acid	1.23	6.82 × 10^−3^	[27]
	Anti-Oxidant	metab_13246	Histidinyl-Hydroxyproline	2.02	7.84 × 10^−3^	[23]
	Anti-Oxidant	metab_9495	N(6)-(Octanoyl)lysine	1.85	7.84 × 10^−3^	[23]
	Anti-Oxidant	metab_18186	6-Hydroxysandoricin	−9.45	6.82 × 10^−3^	[23]
	Oxidative stress	metab_3786	Gamma-Glutamylleucine	10.80	7.84 × 10^−3^	[28]
Immunity	Anticancer	metab_10041	Capsianoside H	2.83	7.84 × 10^−3^	[29]
	Antibacterial	metab_9908	(R)-Roemerine	−2.57	7.84 × 10^−3^	[30]
	Antiviral	metab_8329	1-(3-Furanyl)-6,7-dihydroxy-4,8-dimethyl-1-nonanone	1.58	7.84 × 10^−3^	[31]
	Antiinflammatory/Energy consumption	metab_21033	12a-Hydroxy-3-oxocholadienic acid	1.37	6.82 × 10^−3^	[32]
	Antiinflammatory/Anticancer/Antibacterial	metab_22266	2,3-Dihydroabscisic alcohol	3.54	6.82 × 10^−3^	[33]
	Antiinflammatory	metab_9179	21-Deoxycortisol	1.96	7.84 × 10^−3^	[34]
	Antiinflammatory/Immune responses	metab_23834	2-Hydroxyestrone sulfate	−1.13	6.82 × 10^−3^	[35]
	Antiinflammatory	metab_15702	3-Formyl-6-hydroxyindole	−1.36	6.82 × 10^−3^	[36]
	Antiinflammatory	metab_22693	3-Sulfodeoxycholic acid	1.89	6.82 × 10^−3^	[37]
	Anticancer	metab_12280	5′-Deoxy-5-fluorocytidine	6.87	7.84 × 10^−3^	[38]
	Antiinflammatory	metab_15804	6-Succinoaminopurine	1.30	6.82 × 10^−3^	[39]
	Antibacterial/Anti-inflammatory/Anticancer	metab_5304	Agavoside G	1.45	7.84 × 10^−3^	[40]
	Anticancer/Antibacterial/Anti-inflammatory	metab_1497	Annoglabasin F	−2.24	7.84 × 10^−3^	[41]
	Anticancer/Antiinflammatory/Immunomodulatory/Anti-oxidant	metab_5350	Canarigenin 3-[glucosyl-(1->4)-6-deoxy-alloside]	1.82	7.84 × 10^−3^	[42]
	Immunomodulatory	metab_4216	Dynorphin A (6–8)	2.40	7.84 × 10^−3^	[43]
	Anticancer	metab_20976	Ganoderic acid Mf	1.46	6.82 ×10^−3^	[44]
	Anticancer/Antimicrobial/anti-inflammatory	metab_9442	Isolindleyin	1.46	7.84 × 10^−3^	[45]
	Immune responses	metab_13613	19-Oxoandrost-4-ene-3,17-dione	2.26	7.84 × 10^−3^	[46]
	Antiviral	metab_16386	23-trans-p-Coumaroyloxytormentic acid	4.34	6.82 × 10^−3^	[47]
	Antiinflammatory/Anticancer/Anti-oxidant	metab_11225	Gamma-Glutamyl-S-methylcysteinyl-beta-alanine	−6.43	7.84 × 10^−3^	[48]
	Anticancer	metab_5186	Glutamylproline	1.06	7.84 × 10^−3^	[49]
	Antiinflammatory	metab_19040	Lactosylceramide (d18:1/12:0)	3.38	6.82 × 10^−3^	[50]
Energy	Energy metabolism	metab_11739	Pro-Thr-Thr-Phe	2.11	7.84 × 10^−3^	[51]
	Energy metabolism	metab_5455	Pro-Trp-Phe	1.37	7.84 × 10^−3^	[51]
	Energy metabolism	metab_24676	6,7-Dimethyl-8-(1-D-ribityl)lumazine	2.09	6.82 × 10^−3^	[52]
	Energy metabolism	metab_1299	Ala-Leu-Leu	2.74	7.84 × 10^−3^	[51]
	Energy metabolism	metab_5032	Gly-Leu-Leu	1.98	7.84 × 10^−3^	[51]
	Energy metabolism	metab_2274	Val-Leu-Val-Phe	1.31	7.84 × 10^−3^	[51]
	Energy metabolism	metab_10699	Ala-Ala-Trp-Ile	1.92	7.84 × 10^−3^	[51]
	Energy metabolism	metab_10810	Biliverdin	−1.80	7.84 × 10^−3^	[53]
	Energy metabolism/Antibacterial/Immune reaction	metab_19325	Bisnorcholic acid	3.24	6.82 × 10^−3^	[32]
	ATP enzyme inhibitor	metab_4095	Cyclopiazonic acid	−1.51	7.84 × 10^−3^	[54]
	Energy metabolism	metab_23404	Gamma-L-Glutamyl-L-pipecolic acid	2.61	6.82 × 10^−3^	[51]
	Energy metabolism	metab_11280	Gly-Ile-Val	2.23	7.84 × 10^−3^	[51]
	Energy metabolism	metab_10695	Ile-Ile-Val	1.70	7.84 × 10^−3^	[51]
	Energy metabolism	metab_10648	Ile-Phe-Phe-Thr	2.08	7.84 × 10^−3^	[51]
	Energy metabolism	metab_2709	Thr-Val-Val	1.57	7.84 × 10^−3^	[51]
	Energy metabolism	metab_9753	Trp-Phe	1.78	7.84 × 10^−3^	[51]
	Energy metabolism	metab_12349	Val-Leu-Ser	2.05	7.84 × 10^−3^	[51]
	Energy metabolism	metab_5070	8(R)-HETE	−8.13	7.84 × 10^−3^	[55]
Morphology	Cuticle formation	metab_401	25-Hydroxytachysterol3	1.02	7.84 ×10^−3^	[56]
	Cuticle formation	metab_9436	(2E,8Z)-Decadiene-4,6-diyn-1-yl-3-methylbutanoate	2.47	7.84 × 10^−3^	[57]

**Table 2:** Aquatic adaptation-related pathways (AARPs) identified in aquatic *A. leii*.

Id	Description	FDR
map01523	Antifolate resistance	1.05 × 10^−1^
map00740	Riboflavin metabolism	1.06 × 10^−1^
map04742	Taste transduction	1.06 × 10^−1^
map00600	Sphingolipid metabolism	1.15 × 10^−1^
map00564	Glycerophospholipid metabolism	1.43 × 10^−1^
map00260	Glycine, serine and threonine metabolism	1.50 × 10^−1^
map00230	Purine metabolism	1.92 × 10^−1^
map00140	Steroid hormone biosynthesis	7.86 × 10^−2^
map00982	Drug metabolism-cytochrome P450	8.20 × 10^−2^
map00780	Biotin metabolism	1.03 × 10^−1^

## Data Availability

All metabolomics data generated in this study have been submitted to MetaboLights repository (accession numbers: MTBLS4987).

## Supplementary Materials

The following supporting information can be downloaded at: https://www.mdpi.com/article/10.3390/insects13090823/s1. Figure S1: In the Venn diagram, (A) represents the results obtained by DAM-KEGG-intersection method, (B) represents the results obtained by AARM-KEGG method, as described in detail in Section 2.6. Table S1: The list of DEMs between adult and larval *A. leii*. Table S2: The list of DEMs between adult and larval *L. praetexta*; Table S3. The list of aquatic *A. leii*-specific DAMs between larvae and adults (AARMs). These metabolites did not show differential expression in *L. praetexta*. Table S4: HMDB enriched from *A. leii* DEMs. Table S5: KEGG pathways enriched from *A. leii* DEMs. Table S6: KEGG pathways enriched from *L. praetexta* DEMs. Table S7: KEGG pathways enriched from *A. leii* specific DEMs.
