# Supplementary material for: Global Metabolomics of Fireflies (Coleoptera: Lampyridae) Explore Metabolic Adaptation to Fresh Water in Insects
Source: Insects. 2022 Sep 10;13(9):823. doi: 10.3390/insects13090823 (PMC9503472; doi:10.3390/insects13090823)
Supplement: Supplementary file 1 [file insects-13-00823-s001.zip › Figure S1.pdf]

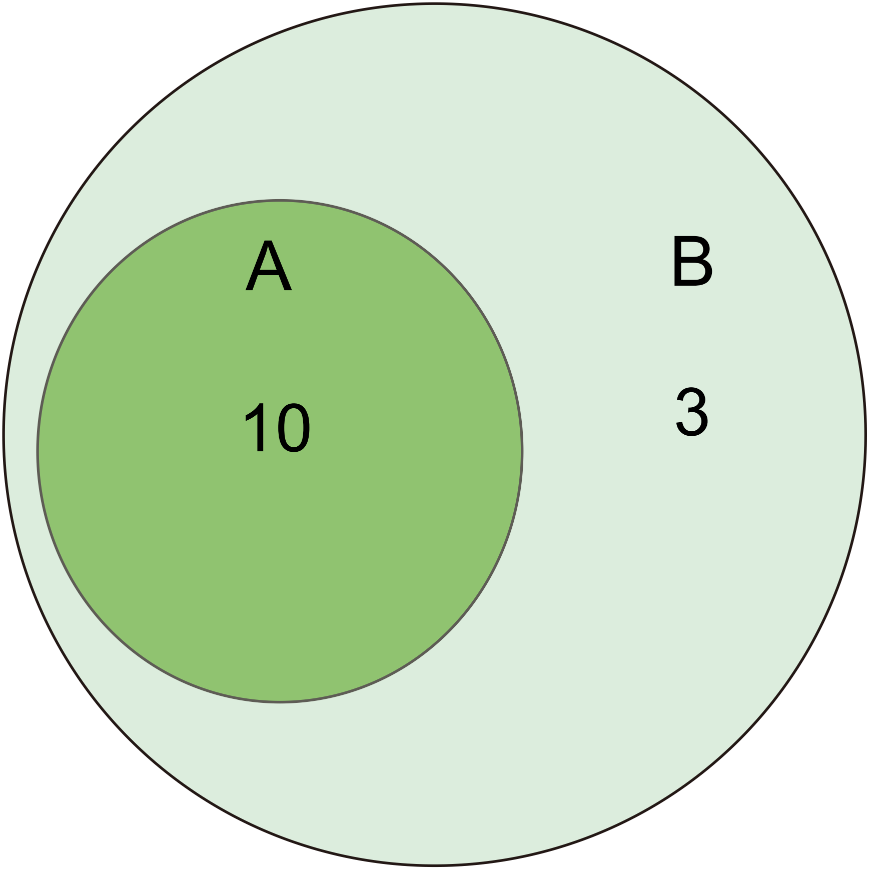
A
B
10
3
